# Haemorrhagic cystitis in haematopoietic stem cell transplantation (HSCT): a prospective observational study of incidence and management in HSCT centres within the GITMO network (Gruppo Italiano Trapianto Midollo Osseo)

**DOI:** 10.3332/ecancer.2014.420

**Published:** 2014-04-10

**Authors:** G Gargiulo, L Orlando, F Alberani, G Crabu, A Di Maio, L Duranti, A Errico, S Liptrott, R Pitrone, S Santarone, C Soliman, A Trunfio, C Selleri, B Bruno, S Mammoliti, F Pane

**Affiliations:** 1 AOU Federico II, Napoli, Italy; 2 Istituto Europeo Oncologia, Milano, 20141, Italy; 3 Istituto di Ematologia Seràgnoli, AOU Sant’Orsola-Malpighi, Bologna, Italy; 4 Ospedale Businco, Cagliari, Italy; 5 Ospedale Ferrarotto, Catania, Italy; 6 AOU Perugia, Italy; 7 AOU Careggi, Firenze, Italy; 8 Ospedale V. Cervello, Palermo, Italy; 9 Ospedale Civile Pescara, Italy; 10 Istituto San Raffaele, Milano, Italy; 11 AO Papa Giovanni XXIII, Bergamo, Italy; 12 A.O.Univ. di Salerno, Italy; 13 Segreteria Naz. GITMO, Italy

**Keywords:** haemmorrhagic cystitis, HSCT

## Abstract

Haemorrhagic cystitis (HC) is a recognised complication in patients undergoing allogeneic haematopoietic stem cell transplantation (HSCT). This study evaluates the incidence and severity of HC in patients undergoing allogeneic HSCT during hospitalisation and within the first 100 days following transplant, looking at the use of prophylaxis, management of HC, outcomes at 100 days post transplant, and to identify any correlations between development of HC and the different conditioning regimens for transplant or HC prevention methods used.

**Results:**

Four hundred and fifty patients (412 adult and 38 paediatric) were enrolled in this prospective, multicentre, and observational study. HC was observed in 55 patients (12.2%) of which 8/38 were paediatric (21% of total paediatric sample) and 47/412 adults (11.4% of total adult sample). HC was observed primarily in the non-related HSCT group (45/55; 81.8%, *p*= 0.001) compared to sibling and myeloablative transplant protocols (48/55; 87.3%; *p*= 0.008) and with respect to reduced intensity conditioning regimens (7/55;12.7%).

In 33 patients with HC (60%), BK virus was isolated in urine samples, a potential co-factor in the pathogenesis of HC. The median day of HC presentation was 23 days post HSCT infusion, with a mean duration of 20 days. The most frequent therapeutic treatments were placement of a bladder catheter (31/55; 56%) and continuous bladder irrigation (40/55; 73%). The range of variables in terms of conditioning regimens and so on, makes analysis difficult.

**Conclusions:**

This multi-centre national study reported similar incidence rates of HC to those in the literature. Evidence-based guidelines for prophylaxis and management are required in transplant centres. Further research is required to look at both prophylactic and therapeutic interventions, which also consider toxicity of newer conditioning regimens.

## Introduction

Haemorrhagic cystitis (HC) is defined as an inflammatory condition of the bladder due to an infectious or non-infectious aetiology resulting in bleeding from the bladder mucosa [1], and which may present with symptoms ranging from mild non-visible microscopic haematuria, to bladder pain, burning on micturition, severe haematuria and potential clot retention and renal failure [2].

In the case of patients undergoing haematological stem cell transplantation (HSCT), there are a wide range of drugs that are implicated in inducing HC, such as cyclophosphamide, ifosfamide, busulphan and thiotepa [1]. As the management of HSCT patients includes prolonged immunesuppression, there is also a susceptibility to develop viral related HC [3]. The consequences of HC impact on the quality of life of HSCT patients, prolonged hospitalisation, increased requirement for transfusion support, and in the most severe cases, this may be a direct cause of patient death.

HC is an acknowledged cause of morbidity and mortality in patients having undergone HSCT [4]. Its reported incidence ranges from 12% to 42% [5–9]) in an allogeneic stem cell transplant setting, with grade 3–4 haematuria in 29%–44% of cases [5, 7, 9].

It may present as an early (within 48–72 h of the conditioning regimen) or late (after 72 h) complication. It is suggested that early onset is related to the effects of the chemo/radiotherapy conditioning regimen [10]. However, various studies have attempted to identify risk factors for later onset HC and correlation is suggested with donor–recipient gender mismatches, stem cell sources, conditioning regimen used, graft versus host disease symptoms, and use of immunosuppressive therapies [5, 6, 11].

Continuous bladder irrigation (CBI) to reduce endothelial drug exposure, and the use of sodium 2-mercaptoethane sulfonate (mesna) have been suggested as prophylactic measures against the development of HC. Results are controversial, with studies demonstrating no difference between use of CBI alone versus mesna alone [12], and others reporting combinations of CBI, mesna, and hydration as being more effective than mesna and hydration alone (incidence of HC 32% versus 50%, respectively) [13].

Similarly, developments in the understanding of the presence and management of viral HC have lead to the call for early diagnosis and treatment to reduce morbidity. Prophylaxis with the flouroquinolone ciprofloxacin has been reported as being safe and effective in reducing BK virus associated HC [14], and in the management of HC cidofovir has become the drug of choice in many cases due to its activity against various viral pathogens [1].

Such advances in prophylaxis and identification of risk factors and early management have lead to a reported reduction in the incidence of HC, however, this remains a real complication for patients undergoing HSCT. The literature reports diverse options for the management of intractable bladder haemorrhage including the use of estrogens [15], prostaglandin bladder installations [16] embolisation, and surgery—such as urinary diversion and cystectomy, which may not be applicable for all patients.

Many of the studies looking at aspects of HC are limited in their ability to provide clear guidance in terms of best practice for this debilitating condition, due to wide variations in factors, such as sample sizes, conditioning regimens for transplant and differing approaches to monitoring, prophylaxis, and management of HC. Research in this field also reports findings from various international settings, however, an understanding of this important clinical issue is lacking within the context of an Italian setting. To begin to address these issues, the GITMO Italian transplant network group performed a prospective, multicentre, observational study across Italy looking at the incidence and severity of HC during HSCT, and factors correlated with its incidence and clinical management and outcomes.

The main objective of the study was to evaluate the incidence and severity of HC in patients undergoing allogeneic HSCT during the period of hospitalisation and within the first 100 days following transplant. Secondary objectives were to analyse the use of prophylactic interventions for HC prevention, identify management strategies for patients developing HC, observe outcomes of HC at 100 days post-transplant, and to identify any correlations between development of HC and the different conditioning regimens for transplant or HC prevention methods used.

## Materials and methods

### Sample

The study was approved by the Ethical Committees both centrally and locally in centres participating in the study.

The study was co-ordinated by the HSCT centre of the Federico II University Hospital in Naples, Italy. All 100 GITMO registered centres for allogeneic transplant were invited to participate in the observational study, of which 30 centres agreed to participate.

All eligible patients were approached by the local study co-ordinator, who explained the objectives of the study, including the observational scope, voluntary participation, and anonymity. At the end of the interview, patients wishing to participate were asked to provide written consent.

### Inclusion criteria

Patients were eligible if they were to undergo allogeneic HSCT, did not have HC before starting the conditioning regime for HSCT, as determined by clinical medical history, including diuresis and urinalysis. Only patients giving written informed consent to participate in the study were includable.

In the case of patients <14 years of age, the parents of the patients were asked to give written consent, however, in the case of patients aged between 14 and <18 years of age, written consent was obtained from both the patient and parents.

### Outcomes

Outcomes for the study included reporting of patient related demographic data, transplant information (type of transplant, source of stem cells, and conditioning regimen), HC related data (prophylaxis, HC incidence, diagnostic tests, severity and use of urinary catheters, other interventions), and outcomes at day 100 post-HSCT. HC was considered as being resolved if the main symptoms were no longer present or absence of haematuria, clots, and/or frustules in the urine, dysuria, or pain on micturition. Data were collected anonymously by experienced haematology nurses or doctors working within each of the transplant centres.

The co-ordinating centre of the study provided a specific manual for the collection and processing of data, providing online support for any difficulties. The protection of the confidentiality of the data were carried out by sending encrypted data accessible only by the co-ordinating centre.

### Statistical analysis

Due to the observational nature of the study, all patients enrolled and considered eligible were included in a descriptive statistical analysis.

Data were prospectively collected within a single database, analysed according to the variables, and was elaborated with the input of a biostatistical expert. Between group analysis was performed using χ2 for the variables. Differences were considered statistically significant with values of *p*< 0.05.

## Results

From 1 January 2011 to 15 February 2012, 450 patients [412 adult (92%) and 38 paediatric (8%)] undergoing allogeneic HSCT in 30 GITMO network transplant centres were enrolled in the study. Patient and treatment characteristics are presented in Table 1.

The median age of patients was 46 years old, prevalently adult (92%) and male (62%). The most frequent diagnosis of patients undergoing HSCT was acute leukaemia (*n*= 282, 63%). Most patients received either a sibling HSCT (45%) or a matched unrelated donor transplant (41.4%), and in 321 cases (71%) a myeloablative HSCT conditioning regimen was used. Thirty nine different conditioning regimen combinations were reported. These were grouped according to the inclusion of drugs implicated in HC development (Table 1). The most commonly used regimens (*n*= 141, 31.4%) included both fludarabine in combination with busulphan or treosulphan, or cyclophosphamide in combination with busulphan or treosulphan (*n*= 93, 20.7%).

### HC prophylaxis

The main interventions for the prevention of HC were hyper-hydration (*n*= 386, 85.8%), alkalinisation of urine (*n*= 280, 62.2%), and the use of mesna in 228 patients (50.6%)—with particular note that 90.2% of patients treated with cyclofosphamide received mesna. The use of quinolone antibiotics was reported in 299 patients (66.4%), as part of prophylaxis for immunosuppression, which was a standard dose (in adult patients—ciprofloxacin (oral) 1 g/day, levofloxacin (oral) 500 mg daily). Table 2shows a summary of HC prophylaxis. Data relating to the use of bladder catheters as prophylaxis for HC were also collected, and used in 53 patients (11.8%).

### Incidence of HC

The development of HC was registered in 12.2% of patients (55/450). A higher incidence was noted in paediatric patients compared to the adult group (21% and 11.4%, respectively) and similar frequency noted between male and female groups (11% and 13%, respectively).

There are five clinical symptoms classified as being correlated with the intensity of HC, referring to the severity of HC and clinical symptoms, according to the classification of [17]:

- grade 0: no haematuria, absence of clinical signs and symptoms.
- grade 1: microscopic haematuria.
- grade 2: macroscopic haematuria.
- grade 3: macroscopic haematuria with the presence of clots.
- grade 4: macroscopic haematuria with the presence of clots, fragments, and urinary retention.

This classification was used to grade the intensity and severity of HC. Within the sample, eight patients experienced grade 4 HC, 21 patients—grade 3, 18 patients—grade 2, and eight patients with grade 1 HC (14.5%, 38.3%, 32.7%, and 14.5%, respectively).

The mean duration of HC was 20 days (range 4–78). HC onset with respect to the day of HSCT infusion, was between 1 and 14 days after infusion in 15 patients, between 15 and 30 days after infusion in 19 patients, between 31 and 60 days after infusion in 17 pz, and between 61 and 100 days after infusion in one in four patients.

In terms of outcomes of HC at day 100 after HSCT reinfusion, 16 patients no longer had HC, whilst in two patients this was less severe. In no cases was exacerbation of HC reported by day 100. However, data are only partially available as 11 patients who experienced HC died before Day + 100, and for a further 23 patients with HC, data were missing regarding the outcome of HC (Table 3).

### Diagnostic interventions in patients with HC

The main diagnostic interventions observed were the performance of urine culture for bacteria (46/55; 83.6%) and/or for BK virus (45/55; 81.8%) and bladder ultrasound (29/26; 52.7%). Urine sample was ‘negative’ in 42 patients and ‘positive’ in four patients, meanwhile the presence of BK virus in the urine was ‘positive’ in 33 patients (30%) and ‘negative’ in 12 (Table 4). BK testing for viral load in the blood, was not standard procedure in many hospitals, and no quantitative data were available for the urine test due to centre variations in test performance and analysis.

### Clinical data of patients with HC

Clinical data of the 55 patients experiencing HC were analysed, with particular attention to the conditioning treatment, intensity, type of HSCT donor, incidence of GvHD, and development of GvHD in relation to donor source.

The conditioning regimens in which the most frequent incidence of HC was observed as absolute values, were in the cyclophosphamide containing regimens (18.6%), Busulphan + Cyclophosphamide containing regimens (14.1%) and Busulphan and Fludarabine containing regimens (15.4%), whilst a high incidence was noted as relative values in the protocols: Busulphan + Cyclophosphamide + Thiotepa + Fludarabine (3/3 patients developing HC—100%), Busulphan + Cyclophsphamide + Thiotepa + Fludarabine (3/8 patients developing HC—37.5%), and Busulphan + Cyclophosphamide + Melphalan (2/6 patients developing HC—33.2%). Table 5provides a summary of conditioning regimens and HC incidence.

In relation to the type of donor source and HC, 10 patients (18.1%) underwent sibling HSCT, 28 (50.9%) matched unrelated donor (MUD) HSCT, 10 (18.1%) haploidentical, and 7 (12.7) cord blood HSCT. With reference to conditioning regime intensity, 48 patients (87.3%) underwent myeloablative HSCT and 7 (12.3%) a reduced intensity conditioning transplant.

A possible correlation between the development of HC and GvHD was hypothesised, however the results demonstrated 28 patients (50.9%) had no signs of GVHD, 12 patients (21.8%) had GvHD grade 1, nine patients (16.3%) grade 2, four patients (7.3%) grade 3, and two patients (3.6%) grade 4.

Finally, patients with HC were stratified according to GvHD and type of donor source, allowing us to observe an incidence of 29% of patients (10/34) who underwent haploidentical HSCT and concomitantly developed HC and GvHD; this incidence is comparable with that of cord blood SCT (7/25; 28%) (Table 6).

### Main therapeutic interventions during HC

The most frequent therapeutic intervention with HC was the introduction of a urinary urethral catheter (UC), positioned in 31 patients (56.3%). The most frequently used was that of a Foley three-way catheter (*n*= 21, 67.7%) with potential also for CBI.

In the nine patients with grade 1 HC, no catheter was positioned, however in 7/19 patients with grade 2 HC, UC was inserted—more often with a two-way Foley urinary catheters (just one patient having a three-way Foley catheter inserted).

In patients with grade 3 HC, a three-way Foley UC was inserted in 15 patients, a two-way Foley UC was inserted in just one patient, and a three-way Couvaliere UC in one patient, while in a further four patients no UC was placed. Finally, patients with grade 4 HC, a two-way Foley UC was positioned in two patients, a three-way Foley catheter in five patients, and no UC was inserted in just one case.

With reference to the timepoint chosen for the insertion of the UC, in 16 patients (51.6%) it was positioned as soon as haematuria began. In 11 patients (35.5%) the catheter was positioned after 24–48 h after the start of haematuria, and in four patients (12.9%) when micturition became difficult due to the presence of clots. The clinical management of patients with HC tends to be under the direct responsibility of the transplant team; the advice of an urologist was reported in four cases (7.3%), whilst for the other 51 patients, this was not requested.

Other therapeutic interventions reported were use of hyper-hydration in 40 patients (72.7%), CBI in 15 patients (27.2%), systemic administration of Cidofovir (*n*= 7;12.7%), hyaluronic acid bladder instillation (*n*= 3; 5.5%); bladder instillation of Cidofovir (*n*= 2; 3.6%); cyctostomy ( n = 1; 1.8%), and intermittent bladder irrigation (*n*= 1; 1.8%).

### Evolution of HC

The study analysed the evolution of HC up to day +100, the end of the observation period, however, data were available for just 32 patients of the 55 who developed HC (Table 3). Complete resolution of HC was reported in 16 patients (29.1%), with two patients experiencing improved HC and two patients with stable HC (3.6%). Eleven patients died before reaching the 100 day observation time-point and one other patient underwent cystectomy. In view of the limited availability of data, it has not been possible to analyse HC outcomes in relation to transplant intensity.

### Possible correlations between occurrence of HC, treatment protocol and clinical condition

Considering the numerous variables related to the clinical conditions and conditioning regimens, it is difficult to hypothesise any correlation with the development of HC. With this in mind, a limited yet in-depth analysis was performed focusing of the following possible relationships (Table 7):

1. HC and the use of cyclophosphamide with or without mesna as prophylaxis (*p*= 0.86);
2. incidence of HC and relationship with age (<18 yrs versus >18 yrs) (*p*= 0.13);
3. incidence of HC and use of myeloablative conditioning regimes versus reduced intensity conditioning regimes—obtaining a statistically significant p value (*p*= 0.008);
4. incidence of HC and type of HSCT donor—sibling versus mismatched donor—obtaining a statistically significant *p*value (*p*= 0.001).

## Conclusion

The study confirms that HC is one of the possible complications of HSCT, with by no means, a negligible incidence (12.2%), which is comparable with published literature [9].

HC requires an intensive therapeutic intervention given that it develops as a more severe form (grades 3 and 4) in 53.8% of cases. In some conditioning regimens incidence of HC is more common—so much so that a synergistic toxicity of different cytotoxic drugs cannot be excluded. The predominant role in the action of polyomavirus in the aetiology of HC is acknowledged within [8], and similar findings have been shown within this study (urinary BK virus, positive in 60% of patients with HC). With the virus being mostly active in immunocompromised patients, and in particular in HSCT mismatched where immunosuppressive therapy is more pronounced.

The majority of prophylactic interventions, such as use of quinolone antibiotics (66.4%), hyper-hydration (85.8%), and use of mesna in cyclophosphamide containing regimens (90.2%), are consolidated in clinical practice. The management of HC, however, appears to be pragmatic. Even if for over 80% of patients, urine culture was performed for bacterial and BK-virus presence, the use of bladder ultrasound to support diagnosis and evaluate HC development is still uncommon (52.7%), as is the demand for specialist urology consultation. Data were collected regarding BK-virus presence and testing, however, the presence or testing of other viruses was not collected, which may be useful in this setting.

The use of bladder catheterisation appears to be uniform within the different centres: in patients with grades 3 and 4 (for which guidelines recommend the insertion of a three-way urinary catheter), only 10% of patients did not have the catheter inserted, whilst a further 5% of patients had two-way catheters inserted and one case using a Couvaliere catheter, as indicated by guidelines [18]. The same guidelines strongly advise against the use of urinary catheters in the absence of specific indications for insertion and therefore this is certainly an aspect of practice to review in cases where urinary catheters were inserted as a prophylactic measure (*n*= 53, 11.8%). It should also be noted that some paediatric centres have emphasised difficulty in finding a three-way catheter with an appropriate calibre for this patient group.

The immunocompromised condition of patients undergoing MUD HSCT is considered globally, taking into consideration the whole care pathway of the patient. Although no data was collected relating to engraftment and onset of HC, we can say that for patients undergoing MUD HSCT, a substantial immunosuppressive therapy is given before and after reinfusion of stem cells, with the development of a prolonged period of immunesuppression, even in the presence of good engraftment, that brings with it a high risk of infections.

In view of the growing increase in the overall number of unrelated donor HSCTs, in which an increased incidence of HC is reported, it is important that each Transplant Programme draws up a specific protocol/guidance for the management of HC, providing specific training and educational courses for healthcare professionals, avoiding experience-based approaches to HC management.

In addition, considering the wide variability of data, further research is required to look at both prophylactic interventions (considering more carefully the toxicity of new conditioning regimens) and therapeutic interventions in HC management.

## Figures and Tables

**Table. 1:** Patient and treatment characteristics.

Variable	*n*= 450
**Median age** age ≥ 18 years old/age < 18 years old	46 years (range 0.5–70) 412 (92%)/38 (8%)
**Gender**: male/female	281 (62%)/169 (38%)
**Diagnosis** Leukaemia: acute/chronic Lymphoma: hodgkin/Nonhodgkin Myeloma Myelodysplasia Myelofibrosis Other	282 (63%)/21 (5%) 26 (6%)/46 (10%) 28 (6%) 23 (5%) 9 (2%) 15 (3%)
**Donor type** Sibling Matched unrelated donor (MUD) Cord blood Haploidentical	205 (45%) 186 (41%) 25 (6%) 34 (8%)
**Conditioning regimen intensity** Myeloablative Reduced intensity conditioning	321 (71%) 129 (29%)
**Conditioning regimen** Cy containing regimen Fl containing regimen Bu/Tr containing regimen Cy & Fl containing regimen Cy & Bu/Tr containing regimen Fl & Bu/Tr containing regimen Cy, Fl & BuTr containing regimen Other	78 (17.3%) 57 (12.7%) 11 (2.4%) 58 (12.9%) 93 (20.7%) 141 (31.4%) 6 (1.3%) 6 (1.3%)

Cy—cyclophosphamide, Fl—fludarabine, Bu/Tr—busulphan/treosulphan.

**Table 2.** Interventions for prophylaxis of HC.

Variable	*N*° patients	*N*° who developed HC	*N*° not developing HC
**Quinolone antibiotics** No antibiotic Ciprofloxacin Levofloxacin Other	53 (11.7%) 125 (27.7%) 174 (38.7%) 98 (20.9%)	4 (7.5%) 17 (13.6%) 18 (10.3%) 16 (16.3%)	49 (92.5%) 108 (86.4%) 156 (89.7%) 82 (83.7%)
**Mesna (total patient sample)** Received mesna Did not receive mesna	217 (48.2%) 233 (51.8%)	28 (12.9%) 27 (11.5%)	189 (87.1%) 206 (88.4%)
**Mesna (only patients receiving Cy — *n*= 233)** Received mesna Did not receive mesna	212 (91%) 21 (9%)	27 (12.7%) 3 (14.3%)	185 (87.3%) 18 (85.7%)
**Hyper-hydration** Yes No	386 (85.8%) 64 (14.2%)	49 (12.7) 6 (9.4%)	337 (87.3%) 58 (90.6%)
**Urine alkalinisation** Yes No	280 (62.2%) 170 (37.8%)	41 (14.7%) 14 (8.2%)	239 (85.3%) 156 (91.7%)
**Insertion of urinary catheter** Yes No	53 (11.8%) 397 (88.2%)	7 (13.2%) 48 (12.2%)	46 (86.8%) 349 (88%)

**Table 3.** Incidence and characteristics of haemorrhagic cystitis.

Variable	*N*° patients
**Total number of patients experiencing HC**	*n*= 55 (12.2% total population)
**Incidence according to age** Mean age of patient was 34yrs (range 10—63 yrs) <18 yrs old ≥18 yrs old	8/38 (21% of paediatric sample) 47/412 (11.4% of adult sample)
**Incidence of HC according to gender** Male Female	32/281 (11% total male population) 23/169 (13% total female population)
**Intensity HC** Grade 1 Grade 2 Grade 3 Grade 4	8 (14.5%) 18 (32.7%) 21 (38.3%) 8 (14.5%)
**Duration** Number of days	Mean 20 (range 4-78)
**Number of days post HSCT when HC developed** Mean 23 (range 2–70) 1–14 days 15–30 days 31–60 days 61–100 days	15 (27.2%) 19 (34.5%) 17 (30.9%) 4 (7.2%)
Outcome of HC at +100 days post HSCT Stable Resolved Improved Worsened Other interventions (cystostomy) No data available	2 (3.6%) 16 (29.1%) 2 (3.6%) 0 (0%) 1 (1.8%) 23 (41.9%)
**Outcomes of HC unavailable at day +100 due to patient death**	11 (20%)

**Table 4.** Diagnostic interventions in patients with HC (*n*= 55).

Diagnostic test	Positive result	Negative result	Not performed
**Urine culture for bacteria**	4 (7.3%)	42 (76.3%)	9 (16.3%)
**Urine culture for presence of BK virus**	33 (60 %)	12 (21.8%)	10 (18.2%)
**Performance of bladder ultrasound Yes No**	29 (52.7%) 26 (47.3%)		

**Table. 5:** Development of HC according to conditioning regimes.

Conditioning regimen in which HC was observed	*N*° patients developing HC/*N*° patients receiving specified regimes
Cy containing regimen	13/70 (18.6%)
Fl containing regimen	5/48 (10.4%)
Bu/Tr containing regimen	0 (0%)
Cy and Fl containing regimen	2/43 (4.7%)
Cy and Bu/Tr containing regimen	13/92 (14.1%)
Fl and Bu/Tr containing regimen	20/130 (15.4%)
Cy, Fl, and BuTr containing regimen	3/3 (100%)

Cy—cyclophosphamide, Fl—fludarabine, Bu/Tr—busulphan/treosulphan

**Table 6.** Haemorrhagic cystitis incidence and correlation with stem cell donor source and GvHD intensity.

Variable	*N*° patients developing HC
**Stem cell source** Sibling Matched unrelated donor Cord Haploidentical	10/205 (18.2%) 28/126 (50.9%) 7/85 (12.7%) 10/34 (18.2%)
**Intensity of conditioning regime** Myeloablative Reduced Intensity Conditioning	48 (87.3%) 7 (12.7%)
**Development of GvHD during HC** None GVHD Grade 1 GVHD Grade 2 GVHD Grade 3 GVHD Grade 4	28 (50.9%) 12 (21.8%) 9 (16.4%) 4 (7.3%) 2 (3.6%)
**Patients with HC and GvHD and relationship with stem cell source** Sibling Matched unrelated donor Cord blood Haploidentical	10/205 (4.8%) 28/126 (15.5%) 7/85 (28%) 10/34 (29%)

**Table 7.** Hypothesised correlation between HC and clinical / therapeutic conditions.

	Development of HC	No HC	Pvalue
**Use of cyclophosphamide with mesna** Cyclofosfamide + mesna as prophylaxis Cyclofosfamide without mesna as prophylaxis	28 4	187 20	0.86
**Age of patient** Age < 18 yrs Age ≥ 18 yrs	8 47	30 365	0.13
**Intensity of condtioning regimen** Myeloablative HSCT Reduced intensity conditioing HSCT	48 7	273 122	0.008
**Transplant type** Sibling HSCT Mismatched HSCT (matched unrelated donor/haploidentical/cord)	10 45	195 200	0.001
